# Reactive oxygen species and cognitive decline: an in-depth analysis

**DOI:** 10.4103/mgr.MEDGASRES-D-25-00114

**Published:** 2026-01-26

**Authors:** Sebastián García Menéndez, Walter Manucha

**Affiliations:** Department of Pathology and Pharmacology, National University of Cuyo, Mendoza, Argentina

Reactive oxygen species (ROS) are a central factor in the pathophysiology of numerous neurodegenerative diseases, including Alzheimer’s disease (AD) and Parkinson’s disease, among others. Excessive ROS leads to oxidative damage to lipids, proteins, and DNA, triggering mitochondrial dysfunction, neuroinflammation, and progressive neuronal death. In this sense, several studies in animal and cellular models have shown that excessive ROS accumulation, together with newly described alterations in key molecular pathways such as mammalian sterile 20-like kinase 1 (MST1), kelch-like ECH-associated protein 1 (KEAP1)/phosphoglycerate mutase family member 5 (PGAM5)/apoptosis-inducing factor mitochondria-associated 1 (AIFM1), and dynamin-related protein 1 (Drp1), promotes the degeneration of neuronal synapses, gradual and inexorably compromising cognitive functions. Therefore, there is special interest in developing strategies based on mitochondria-targeted antioxidants and gene-based methodologies focusing on enzymes such as peroxiredoxin 3 (PRDX3), which reveal promising therapeutic approaches and, potentially, preventive measures. Finally, the present manuscript proposes an original perspective on the most relevant recent literature. It delves into the main aspects of ROS production, its impact on mitochondrial dysfunction and neuroinflammation, and various interventions to mitigate oxidative stress and prevent cognitive decline.

Population aging involves an increase in the prevalence of neurodegenerative diseases such as AD and Parkinson’s disease, both characterized by progressive neuronal loss and cognitive or motor dysfunction.^1^ Despite therapeutic advances, current strategies often address symptoms rather than offering a definitive cure, and many are accompanied by highly unpleasant side effects.^2^ This highlights the crucial need for further research to develop more effective and less invasive treatments.

Among the multiple pathophysiological mechanisms described, oxidative stress emerges as a pivotal factor in the majority of neurodegenerative disorders. Due to its high oxygen consumption and abundant content of polyunsaturated lipids, the brain is particularly susceptible to ROS-mediated damage.^3^ Furthermore, excessive ROS production and insufficient endogenous antioxidant capacity promote mitochondrial dysfunction, neuroinflammation, and neuronal death.^4^

Given this background, the main objective of the present review is to deepen our understanding of the most relevant aspects of excessive ROS production and its impact on neurodegeneration. We also discuss the relationship with mitochondrial dysfunction by describing associated cell-death pathways. Finally, we analyze current and future therapeutic strategies that aim to regulate ROS levels and reinforce the antioxidant capacity of certain active principles.^5^,^6^

**Generation of ROS and oxidative stress in neurodegenerative diseases:** ROS, a group of species including the superoxide radical (O_2_^•−^), hydrogen peroxide (H_2_O_2_), and the hydroxyl radical (^•^OH), among others, play a pivotal role in neurodegenerative diseases. Under physiological conditions, these molecules participate in intracellular signaling pathways. They are regulated by enzymatic (superoxide dismutase, catalase, and glutathione peroxidase) and non-enzymatic (reduced glutathione and vitamin C) antioxidant systems.^6^,^7^

However, alterations in mitochondrial oxidative phosphorylation, the presence of misfolded proteins, and the activation of pro-oxidant enzymes (e.g., nicotinamide adenine dinucleotide phosphate reduced-NADPH-oxidase) significantly increase ROS generation.^1^ This ROS overload profoundly impacts the brain, leading to early dysfunction in the mitochondrial respiratory chain in neurons and glial cells, fostering excessive ROS, and correlating with amyloid-β accumulation in AD.^7^,^8^ This situation is one of the main reasons many studies link elevated ROS with deficits in memory and learning. Oxidation of lipids and synaptic proteins in AD diminishes neuronal plasticity, resulting in a marked progressive loss of synapses.^9^ Likewise, in Parkinson’s disease, ROS overload damages the substantia nigra, reducing dopamine levels and disrupting the nigrostriatal, mesolimbic, and mesocortical pathways, thereby exacerbating the motor and cognitive symptoms characteristic of this disease.^6^

Moreover, oxidative stress has been observed to play a significant role in the progression of neurodegenerative diseases. It facilitates the aggregation of pathological proteins (α-synuclein in Parkinson’s disease, amyloid-β in AD), perpetuating a vicious cycle of oxidative damage and protein dysfunction.^8^,^10^ The redox imbalance also impacts mechanisms of autophagy, proteasomal degradation, and DNA repair capacity, thus worsening the neurodegenerative process.^2^

**Damage and neuroinflammation:** Mitochondria, the cell’s powerhouses, produce ROS as by-products of aerobic metabolism and sustain direct damage from ROS overproduction.^5^ This crucial role of mitochondria in ROS production and damage is a key area of study in neurodegenerative diseases. Such injury disrupts the permeability of the inner mitochondrial membrane and impairs oxidative phosphorylation, reducing adenosine triphosphate synthesis.^9^ In addition, it promotes cytochrome C release and caspase activation, leading to further neuronal apoptosis.^6^,^7^

Additionally, MST1/KEAP1-PGAM5/AIFM1 pathway and other regulated cell-death pathways (oxeiptosis and ferroptosis) are exacerbated by high ROS levels, contributing to neuronal death.^8^,^10^,^11^ In this sense, the latest research underscores the critical role of the KEAP1/PGAM5/AIFM1-mediated oxeiptosis pathway in neuronal cell death, offering insights into potential therapeutic targets for mitigating neurodegeneration in AD.

An inflammatory microenvironment amplifies the effects of oxidative stress. When microglia and astrocytes detect ROS and misfolded peptides, they begin secreting pro-inflammatory cytokines, such as interleukin-1β and tumor necrosis factor-α,^9^,^12^ which in turn further increase ROS production via NADPH oxidase.^5^

This cascade damages AD’s critically important blood–brain barrier, increasing synaptic dysfunction and facilitating amyloid-β accumulation.^3^ This underscores the urgent need for effective treatments. Similarly, in Parkinson’s disease, excessive glial cell activation accelerates neurodegeneration in the substantia nigra.^6^

Notably, nano-pharmacology offers unprecedented opportunities to mitigate mitochondrial damage and the inflammatory response simultaneously by allowing precise drug targeting to microglia, astrocytes, and other involved cells.^10^,^13^ In this regard, nanoparticles have been developed to deliver antioxidant and anti-inflammatory agents directly to affected brain areas, thereby selectively reducing ROS production and safeguarding neuronal function.

**Therapeutic strategies targeting oxidative stress and cognitive decline:** The development of nanoenzymes and compounds with superoxide dismutase and catalase-type capabilities presents a promising avenue for neutralizing ROS within mitochondria. Nanoformulations such as 3-carboxypropyl-triphenyl-phosphonium bromide (TPP)-modified (Cu_2_-xSe Cu^2+^-xSe-TPP) demonstrate high affinity for the mitochondrial membrane and mimic superoxide dismutase/catalase activities, effectively reducing ROS production and protecting against mitochondrial dysfunction.^5^ This advancement instills optimism for the future of mitochondrial therapies.

Nanostructure-based therapies have shown remarkable potential in modulating microglial polarization toward an anti-inflammatory macrophage M2 phenotype. This not only promotes the phagocytosis of toxic peptides such as amyloid-β but also significantly improves cognition and slows the progression of AD in murine models.^5^ These promising results offer hope for the potential impact of these therapies on neurodegenerative diseases.

Another promising line of research is the overexpression of antioxidant enzymes. In a paraquat-induced Parkinson’s disease model, the administration of PRDX3 using a cell-penetrating peptide vector demonstrated robust protective effects in the substantia nigra, effectively preventing dopaminergic neuron death.^6^ These findings provide reassurance about the potential of restoring mitochondrial redox homeostasis by modulating antioxidant enzymes such as PRDX3 to mitigate cellular senescence processes and reduce motor and cognitive symptoms.

The importance of the AMPK/Drp1 pathway in mitochondrial dynamics and neuronal energy homeostasis cannot be overstated. A recent study in AD models has demonstrated that treatment with ginsenoside Rg1 modulates AMPK by increasing its phosphorylated form and decreasing Drp1 phosphorylation, favoring mitochondrial fusion and improving bioenergetic efficiency.^4^

The result is a decrease in ROS levels, increased adenosine triphosphate production, and rescue of synaptic dysfunction, leading to improved spatial memory.^4^ These promising results from phytotherapeutic and ethnomedicinal interventions not only demonstrate their potential but also inspire optimism for the future of managing neurodegenerative disorders.

Furthermore, various preclinical models have demonstrated the efficacy of plant extracts and naturally occurring compounds with antioxidant properties. For instance, ginseng and its active components, including ginsenoside Rg1, have been reported to reduce amyloid-β accumulation and enhance mitochondrial function.^4^ Other compounds, such as L-cysteine, have demonstrated neuroprotective effects by mitigating endoplasmic reticulum stress and restoring mitochondrial dynamics.^9^ Moreover, by reducing ROS, these compounds improve cognition and decrease apoptosis in AD models.^9^,^12^ Another complementary approach involves vitamin D, which regulates calcium homeostasis and exerts immunomodulatory and antioxidant effects in the brain. Its ability to modulate proinflammatory cytokines and support neuroprotective pathways has led to growing interest in its potential to slow cognitive decline in conditions such as AD, Parkinson’s disease, and multiple sclerosis. Through regulation of glial cell activity and critical gene networks (both nuclear and mitochondrial), vitamin D may lessen ROS-mediated damage and reinforce endogenous defense systems. Although additional clinical trials are needed, these findings highlight vitamin D supplementation as a potentially valuable adjunct to mitochondrial antioxidants, nanoformulations, and gene therapies to mitigate neuroinflammation and oxidative stress.^14^

**Figure 1:**
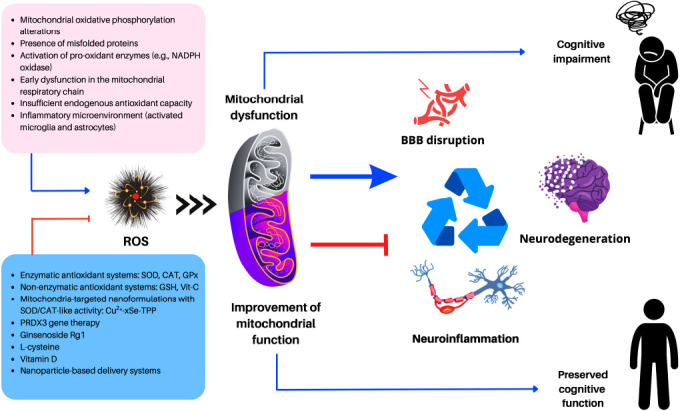
Regulation of ROS levels and their impact on cognitive function. Excessive accumulation of ROS, triggered by mitochondrial oxidative phosphorylation alterations, the presence of misfolded proteins, activation of pro-oxidant enzymes such as NADPH oxidase, early dysfunction in the mitochondrial respiratory chain, insufficient endogenous antioxidant capacity, and an inflammatory microenvironment characterized by activated microglia and astrocytes, leads to mitochondrial dysfunction, neuroinflammation, BBB disruption, and neurodegeneration—ultimately contributing to cognitive impairment. However, there is hope in the form of therapeutic interventions. These include mitochondria-targeted nanoformulations that mimic SOD and CAT activity (e.g., Cu²^+^-xSe-TPP), gene therapy with PRDX3, the use of the phytocompound ginsenoside Rg1, the antioxidant amino acid L-cysteine, vitamin D supplementation, and nanoparticle-based delivery systems. These interventions restore mitochondrial function and preserve cognitive performance, offering hope for potential treatments. Created with BioRender.com. BBB: Blood-brain barrier; CAT: catalase; GSH: glutathione; NADPH: nicotinamide adenine dinucleotide phosphate; PRDX3: peroxiredoxin 3; ROS: reactive oxygen species; SOD: superoxide dismutase; TPP: 3-carboxypropyl-triphenyl-phosphonium bromide; Vit-C: vitamin C.

Evidence suggests that combining mitochondria-directed antioxidants, which are antioxidants specifically targeted to the mitochondria, gene therapy, and the modulation of signaling pathways could yield more robust outcomes in preventing and treating cognitive decline.^2^,^3^ Furthermore, nanoparticle-based strategies have emerged as an up-and-coming alternative, given their ability to enhance the stability of antioxidant compounds, more precisely target mitochondria, and additionally potentiate neuroimmune modulation by effectively crossing the blood-brain barrier.

Recent nanopharmacology studies have shown that various nanoformulations attenuate microglial inflammation, protect neurons against oxidative stress, and restore energetic homeostasis.^10^,^13^ These findings suggest a promising future in therapeutics, where mitochondrial targeting, gene therapy, and oxidative stress biomarkers could significantly improve early intervention and clinical response monitoring. The use of oxidative stress biomarkers, such as 4-hydroxynonenal and 8-hydroxy-2’-deoxyguanosine, not only offers diagnostic tools for early intervention but also provides a means for evaluating therapeutic response. However, challenges such as optimizing drug bioavailability across the blood–brain barrier and tailoring doses to minimize adverse effects remain. It is also crucial to further explore the impact of these therapies on the immune system and the gut microbiota, whose relationship with neurodegenerative diseases has become increasingly significant.^9^,^12^ It could open up new avenues for research and collaboration.

**Conclusions and perspectives:** Oxidative stress, arising from excessive ROS levels and mitochondrial dysfunction, is one of the main drivers of cognitive decline associated with neurodegenerative diseases such as AD and Parkinson’s disease. The effectiveness of strategies involving mitochondrial antioxidants, gene therapies, and signaling pathway modulators in reversing or slowing neurodegeneration has been well-demonstrated, providing reassurance about the progress in the field.

Current research confirms the potential of agents such as Cu^2+^-xSe-TPP, PRDX3, or ginsenoside Rg1 to restore mitochondrial function, reduce ROS, and safeguard synaptic function. As a graphical overview, the figure illustrates the central pathways that determine ROS levels, their effects on cognitive function, and their modulation and impact on neuroprotection.

However, further clinical trials and optimization of drug administration methods are urgent. Overcoming obstacles such as the blood–brain barrier is crucial. Ultimately, modulating oxidative stress is emerging as a pivotal therapeutic axis for preventing or mitigating cognitive decline, and your continued research is vital in the fight against neurodegenerative disorders.

*The present work was funded by grants from the Research and Technology Council (SIIP 2022-2025) of National University of Cuyo, Mendoza, Argentina, as well as from the National Agency for the Promotion of Research, Technological Development and Innovation ANPCyT FONCyT (Grant no. PICT 2020 Serie A 4000) (to WM).*
